# A Case of Epithelioma of the Gums, with a Few Remarks

**Published:** 1888-06

**Authors:** Frank Lankester


ARTICLE VII.

### A CASE OF EPITHELIOMA OF THE GUMS, WITH A FEW REMARKS.

BY FRANK LANKESTER, L. R. C. P., M. R. C. S., L. D. S., ENG.

The following case came under my notice some few months ago at the National Dental Hospital. Though a purely surgical affection, it, nevertheless, had a dental origin, and will therefore, I feel sure, prove of considerable interest and be of some practical value to the members of our profession. The patient, on presenting himself, complained simply of a "rather sore gum." I elicited the following history:—George H., aged 55, a coachman, with an excellent family history, said he had always enjoyed thoroughly good health himself. During the last twenty years he had gradually lost most of his teeth, so that in the upper jaw only the two centrals and the right canine now remained. Up to about a year ago, however, the left canine root was *in situ*, but covered over by a vulcanite artificial denture, which he had been wearing for several years previously. He did not remember having had any trouble whatever with this left canine root until about eight or nine months ago, when, without any very obvious cause, it became somewhat loose; whereupon he began to "work it about" with his fingers, so that, two or three months later, the root came away altogether. The socket, however, never healed up in the usual way; on the contrary, the vacant space caused by the loss of the tooth had been steadily, though very slowly, enlarging ever since. During the last month the ulcerative process had progressed more rapidly; there had been more tenderness and pain, together with a fœtid discharge. He had previously experienced very little pain. After the root came away, the artificial canine no longer fitted closely up to the gums, so that, three weeks previously to my seeing him, he had consulted a dentist; but the latter merely added some more vulcanite, so as to form the usual artificial gum. He thus covered up the ulcerating surface, and boxed in the foul discharge, fondly hoping that the "rest" thus afforded would enable the gums to resume their normal condition. A week later, he went to a dispensary, where he was given some liniment, which was, of course, worse than useless in his case. He next sought our advice, but not until three valuable weeks had been lost, owing to the culpable ignorance or surgical inexperience on the parts of the dentist and the "dispensing doctor." The disease was so far advanced that there could have been no difficulty whatever in diagnosing or at least suspecting it, yet the patient was not warned of his condition nor even sent elsewhere for another opinion. And yet men ask what is the use of a dentist knowing anything about general surgery.

*On examination*, patient had a well marked sallow and cachectic appearance. There was a slight fullness and puffiness over the left cheek, whilst, beneath the angle of the lower jaw, was to be felt a hard glandular mass about as large as a walnut.

Within the mouth, the left central was very loose, and the right partially so. In the situation of the left canine was a hole extending upwards towards the antrum, and nearly large enough to admit the tip of the little finger. The surface was "dirty" and ulcerating, as was also that of the gum in the immediate neighborhood, whilst as far back as the tuberosity the surface of the gum was much thickened and nodular, though not actually ulcerating throughout its whole extent. The buccal mucous membrane was becoming involved by the disease, and the tissues all round the growth were much indurated. There was not much discharge, but the breath was very offensive. Patient said he was losing flesh. There was not the least doubt as to its epitheliomatous nature, so I sent him on to St. Thomas's Hospital, warning him that he must not play with the disease any longer. He was at once admitted, under the care of Mr. Clutton, Two or three days later, patient was put under chloroform, and Mr. Clutton removed the lower half of the left superior maxilla, whereupon it was found that the growth extended over to the right side of the septum nasi—a very serious discovery, pointing strongly to a probable recurrence of the disease, owing to the increased difficulty in getting rid of all the tissues involved, besides greatly prolonging and increasing the severity of the operation; the lymphatic glands were left for subsequent removal, when the patient should have recovered from the shock of the operation.

For about a week patient progressed most favorably, when pyæmia suddenly set in, to which he rapidly succumbed. The disease was so far advanced that, had the man recovered from the operation, it would have probably recurred, but the operation would have prolonged his life, and would have prevented the endurance of much subsequent suffering.

This unfortunate termination of the case was much to be regretted, but it does not alter the fact that, had the nature of the disease been recognized in the first instance, the man would probably have been alive and well now. For the operation would have been a comparatively small affair, though, of course, a free excision would have been necessary. There would have been less danger, too, of pyæmia, &c., and the man would have been in a better state of health generally.

In cases like this it is, of course, obviously all-important that an early and correct diagnosis be made. The earlier the stage of the disease, the more difficult necessarily does the diagnosis become, and, therefore, the greater the need of being thoroughly well acquainted with the disease, its diagnostic points, and its serious prognosis.

Epithelioma of the gums, primarily, is somewhat rare, but when it does occur, it will probably be in connection with some long continued irritation, such as that caused by an ill fitting plate, a carious stump, &c., nor should we forget that the sharp-pointed edge of a tooth is one of the most frequent causes of cancer of the tongue. Thus, probably, the dentist will be the first man called upon to give relief in these cases; and if he be able, without delay, to correctly diagnose, and advise as to the treatment of, an epitheliomatous ulcer when present, it will be a great source of subsequent satisfaction and value to both dentist and patient, but especially to the latter.

Side by side with this case we may place an exactly similar one mentioned by Sir John Tomes in the third edition of his "Dental Surgery" (p. 737). Here, also, the disease originated "in the edges of the socket of a dead tooth." But it was early recognized, and the tuberosity, with the hard and soft palates, were excised with the satisfactory result that there has been no recurrence, "a success due to its early recognition."

What, then, are the chief points that will help us to this end? We should regard with very grave suspicion any ulcerated surface in the mouth, with induration of the surrounding tissues, occurring in a male over forty years of age, and which has not yielded at all to specific or other treatment, but in spite of it continues slowly but surely to increase in extent, even after any possible exciting cause has been removed. We should notice the general appearance of the patient, and inquire carefully as to whether there be any family predisposition to cancer, and whether the patient be losing flesh. Examine carefully if any of the lymphatic glands be involved, and lastly, we may call the microscope to our aid. Scrape the surface of the ulcer and mix with a drop of water; place on a slide and examine carefully under a moderate power. If the ulcer be epitheliomatous in nature, you will most probably find here and there aggregations of epithelial cells, the so-called "birds' nests" or "nest cells." These, then, are the chief points, and it surely behooves us all to bear them in mind.—*London Dental Record.*

